# Increased Mortality Associated with Amiodarone Compared to Other Antiarrhythmic Drugs in New-Onset Atrial Fibrillation

**DOI:** 10.3390/jcm14041168

**Published:** 2025-02-11

**Authors:** Yun Gi Kim, Hyoung Seok Lee, Hoseob Kim, Mina Kim, Joo Hee Jeong, Yun Young Choi, Jaemin Shim, Jong-Il Choi, Young-Hoon Kim

**Affiliations:** 1 Division of Cardiology, Department of Internal Medicine, Korea University College of Medicine, Korea University Anam Hospital, Seoul 02708, Republic of Korea; tmod0176@gmail.com (Y.G.K.); leehyoungseok0511@gmail.com (H.S.L.); jessica0115@naver.com (J.H.J.); yych60@naver.com (Y.Y.C.); jaemins@korea.ac.kr (J.S.); yhkmd@korea.ac.kr (Y.-H.K.); 2 Department of Data Science, Hanmi Pharm Co., Ltd., Seoul 05545, Republic of Korea; hoseob.kim@hanmi.co.kr (H.K.); mina.kim92@hanmi.co.kr (M.K.)

**Keywords:** amiodarone, antiarrhythmic drugs, atrial fibrillation, mortality

## Abstract

**Background and Aims:** Antiarrhythmic drugs (AADs) are the primary treatment for maintaining sinus rhythm in patients with atrial fibrillation (AF). While amiodarone is mainly used in patients with structural heart disease, its effects on all-cause mortality compared to other AADs remain unclear. **Methods:** This study utilized nationwide healthcare insurance data involving patients with new-onset AF from 2013 to 2019. We identified patients who were prescribed with AADs ≥ six months within the first year of diagnosis (medication possession ratio ≥ 0.5). All-cause mortality was assessed between amiodarone and other AAD users up to three years post-AAD-prescription. **Results:** Among 770,977 new-onset AF patients, 12,243 were amiodarone users and 33,036 were prescribed with other AADs. Significant differences in mean age and prevalence of medical conditions such as heart failure, myocardial infarction, chronic kidney disease, diabetes, and dyslipidemia were noted. After propensity score matching, 12,017 amiodarone users were compared to an equal number of other AAD users with similar baseline characteristics. Multivariate analysis indicated a 2.9-fold increase in all-cause mortality for amiodarone users (hazard ratio = 2.88; 95% confidence interval = 2.64–3.15; *p* < 0.001). This increased risk was more pronounced among women compared with men (hazard ratio = 3.38 vs. 2.56; *p* for interaction = 0.004). Amiodarone was associated with increased mortality in AF patients with heart failure and myocardial infarction. **Conclusions:** Amiodarone, compared with non-amiodarone AADs, was associated with increased risk of all-cause mortality in AAD-naive new-onset AF patients. Increased all-cause mortality associated with amiodarone was consistent throughout various subgroups. Significant interaction was observed with the sex category, with women being more vulnerable to amiodarone.

## 1. Introduction

The incidence and prevalence of atrial fibrillation (AF) is anticipated to increase rapidly mainly due to aging population and advancement in diagnostic tools such as wearable devices [1]. In addition to anticoagulation therapy, rhythm control therapy is an important part of holistic AF management [2]. Antiarrhythmic drugs (AADs) that act on ion channels are used to maintain sinus rhythm and relieve symptoms in AF patients. However, the clinical benefit of AADs, especially regarding hard clinical outcomes such as death, stroke, or heart failure hospitalization, remains unclear. The AFFIRM trial revealed that rhythm control strategy did not improve all-cause mortality in AF patients [3]. In patients equal or older than 65 years, rhythm control strategy was associated with significantly higher all-cause mortality [3]. In the trial, amiodarone was the most frequently used AAD (37.5% as an initial drug and 62.8% as a drug used at any time). In contrast, rhythm control strategy, when started early after diagnosis of AF, was associated with significantly reduced risk of death, stroke, or serious adverse events in the EAST-AFNET 4 trial [4]. The major difference between the two trials, in addition to duration of AF, was the drugs used for rhythm control strategy. In the AFFIRM trial, the most frequent AADs used were amiodarone (37.5%) and sotalol (31.2%) [3]. In contrast, flecainide (35.9%) followed by amiodarone (19.6%) was the most frequent AAD used in the EAST-AFNET 4 trial [4]. The difference in type of AADs, short AF duration, and utilization of AF ablation procedure in the EAST-AFNET 4 trial can be the cause of better clinical outcome in the rhythm control group.

The antiarrhythmic effect is reported to be higher in amiodarone compared to other AADs such as propafenone or sotalol [5,6,7]. However, amiodarone is known for various cardiac and extracardiac toxicities such as pulmonary fibrosis, thyroid function abnormalities, non-alcoholic steatohepatitis, or corneal deposits [8,9,10,11,12]. Despite its potential toxicities, amiodarone is widely used in clinical practice for new-onset atrial fibrillation mainly due to its superior efficacy and broad applicability. For example, dronedarone is contraindicated in heart failure patients (ANDROMEDA trial) and Class IC drugs (encainide or flecainide) were associated with increased mortality in acute myocardial infarction patients (CAST-II trial) [13,14]. Since AF is often accompanied by heart failure and atherosclerotic cardiovascular disease, amiodarone can be preferred over other drugs to avoid any adverse potential interactions. However, the enrolled patients in the ANDROMEDA and CAST II trial were not AF patients, but rather they were severe heart failure and acute myocardial infarction patients. Therefore, the impact of amiodarone on overall mortality in comparison to other AADs is not fully revealed. We conducted this analysis to evaluate whether amiodarone is associated with increased mortality compared with other AADs in AF patients undergoing rhythm control.

## 2. Materials and Methods

### 2.1. Study Cohort

Data from the Korean National Health Insurance Service (K-NHIS) were used for this study. All citizens of the Republic of Korea are mandatory subscribers of the K-NHIS which is the exclusive medical insurance system managed by the Korean government. Due to its exclusive nature, the entire population of the Republic of Korea is represented by the K-NHIS. For medical research purposes, the K-NHIS can be utilized upon approval from the official K-NHIS review committee (https://nhiss.nhis.or.kr/). This study was approved by the Institutional Review Board of Korea University Medicine Anam Hospital and the official review committee of K-NHIS. Written informed consent was waived by the Institutional Review Board of Korea University Medicine Anam Hospital since this study was based on retrospective analysis. The legal regulations of the Republic of Korea and the ethical guidelines of the 2013 Declaration of Helsinki were strictly adhered to throughout the study. To conduct this study, the data was accessed between 1 July 2020 and 31 December 2021.

The K-NHIS database contains claims of International Classification of Disease, 10th edition (ICD-10) diagnostic codes and prescription history of legally approved drugs. If a given subscriber dies due to any cause, he or she is automatically excluded by the K-NHIS system. Therefore, the date of death is clearly identifiable for all subscribers. Biennial nationwide health screening is offered to its subscribers which comprises (i) medical measurements such as blood pressure, body weight, and stature; (ii) self-report questionnaires regarding smoking status, alcohol consumption habits, and exercise level; and (iii) laboratory tests such as complete blood cell counts, serum creatinine level, liver function tests, lipid profiles, and fasting blood glucose.

### 2.2. Diagnosis of AF

People who were diagnosed with new-onset AF during 1 January 2013 and 31 December 2019 were enrolled in this study. The diagnosis of AF was based on claim of ICD-10 codes for AF (I480, I481, I482, I489; Supplementary Table S1). To rule out prior diagnosis of AF, we excluded people who were diagnosed with AF during 1 January 2010 and 31 December 2012. Therefore, AF cases analyzed in this study are new-onset AF.

### 2.3. Prescription of AAD

We included those who started an AAD within one year after diagnosis of AF. The use of AADs was identified through prescription history stored in the K-NHIS database. All AADs available in the Republic of Korea were evaluated which includes amiodarone, dronedarone, sotalol, flecainide, propafenone, and pilsicainide (Supplementary Table S2). Quinidine was not included since it is scarcely prescribed to treat AF. Medication possession ratio (MPR) was defined as (prescribed days of a certain AAD/365 days starting from the first prescription date) × 100 (%). Equal or more than 50% of MPR was the inclusion criteria of this study. In other words, if a screened new-onset AF patient was prescribed with AADs for equal or more than 183 days during the first year of prescription, he or she was classified as an AAD user and was included in the study. We defined MPR ≥ 0.5 to achieve a high mean MPR in enrolled patients. Since the half-life of amiodarone is long, some side effects can happen several months after discontinuation, which makes difficult to define which AAD is responsible for death in patients who had alternating AAD treatment. Therefore, people who were prescribed multiple AADs or changed their AAD (amiodarone to other AADs or vice versa) within one year after taking AAD were excluded. Clinical follow-up was three years starting from the date of AAD prescription, and crossover to a different AAD during first year of follow-up was an exclusion criterion. Those who did not use AADs within one year of AF diagnosis was not included in this study.

### 2.4. Definitions

Identification of prior medical history such as hypertension or diabetes mellitus was based on claim of ICD-10 codes and medical measurements within three years prior to the diagnosis of new-onset AF. ICD-10 codes for each disease are summarized in Supplementary Table S3. Hypertension was diagnosed if ICD-10 codes for hypertension were claimed or measured systolic or diastolic blood pressure were equal or higher than 140 and 90 mmHg, respectively. Diabetes mellitus was identified based on ICD-10 codes or measured fasting blood glucose (if equal or higher than 126 mg/dL). Dyslipidemia was based on prior claims of ICD-10 codes. Chronic kidney disease was diagnosed if estimated glomerular filtration rate was under 60 mL/min/1.73 m^2^ or ICD-10 codes for chronic kidney disease were claimed. Heart failure, myocardial infarction, and thyroid disease was diagnosed based on ICD-10 codes.

Baseline demographics were based on nationwide health screening within three years of new-onset AF diagnosis. Alcohol consumption status was classified as follows: (i) non-drinkers: 0 g per week; (ii) mild to moderate drinkers: 0 g to 210 g per week; and (iii) heavy drinkers: 210 g or more per week. Smoking history was defined as follows: (i) never-smokers: those who smoked < 100 cigarettes in their lifetime; (ii) ex-smokers: people who smoked more than 100 cigarettes in their lifetime but had not smoked within one month of enrollment (diagnosis of new-onset AF); (iii) current smokers: people who smoked more than 100 cigarettes and continued to smoke within one month of enrollment. Performing one or more high-intensity (such as running, climbing, or intense bicycle activities) or moderate-intensity (such as walking fast, tennis, or moderate bicycle activities) exercise per week was defined as having a regular physical activity. The robustness of these definitions was validated in our prior studies [15,16].

### 2.5. Primary Outcome Endpoint

All-cause death was the endpoint of this study. The occurrence of death is readily identifiable through the K-NHIS database. The subscribers of the K-NHIS lose their subscriber status immediately after the death certificate is reported to the Korean government. Except for emigration, there were no losses to follow-up. However, those who emigrate lose their subscriber status and therefore are censored at the time of emigration.

### 2.6. Statistical Analysis

In this study, we tested the hypothesis that as compared to other antiarrhythmic drugs, amiodarone is associated with significantly increased risk of all-cause death in patients with new-onset atrial fibrillation undergoing pharmacologic rhythm control therapy. Continuous variables were compared with the Student’s *t*-test and categorical variables with the Chi-square test. Kaplan–Meier survival curve analysis with log-rank t-test was performed to depict and compare cumulative incidence of all-cause death between groups. Cox regression analysis was used to calculate hazard ratio (HR) and 95% confidence interval (CI). Five multivariate-adjusted models were analyzed: (i) model 1: adjusted for age and sex; (ii) model 2: adjusted for model 1 plus hypertension, diabetes mellitus, dyslipidemia, chronic kidney disease, heart failure, myocardial infarction, and thyroid disease; (iii) model 3: adjusted for model 2 plus alcohol, smoking, BMI, regular exercise, estimated glomerular filtration rate, and total cholesterol; (iv) model 4: adjusted for model 2 plus type of AAD as a time-varying covariate; and (v) model 5: adjusted for model 4 plus performance of catheter ablation for AF during the screening and follow-up period. Variables such as alcohol, smoking, BMI, regular exercise, estimated glomerular filtration rate, and total cholesterol were measured by self-questionnaire during nationwide health screening. Since a substantial proportion of patients did not undergo nationwide health screening, there were patients with missing values, and these patients were not included in model 3. For this reason, our main multivariate model was model 2. Participants who changed their type of AAD within one year after enrollment were excluded but those who changed their AAD after one year of use were included in the study with adjustment as a time-varying covariate. Propensity score matching (PSM) was performed in addition to multivariate adjusted analysis. One to one propensity score matching (PSM) analysis was performed to minimize the differences between two groups. Multiple logistic regression analysis which included covariates of age, sex, smoking, alcohol, regular exercise, income, diabetes mellitus, hypertension, dyslipidemia, heart failure, myocardial infarction, chronic kidney disease, thyroid disease, and stroke was performed to calculate propensity scores. Greedy method within a caliper of 0.1 was used for matching since this value has been shown to eliminate over 90% of the bias in the observed confounders. Calculation of absolute standardized differences in the baseline characteristics was performed to assess the success of PSM. Insignificant difference in each covariate was defined as absolute standardized difference of <0.1. All tests were two-tailed, and *p* values equal or less than 0.05 were considered as statistical significant. SAS version 9.4 (SAS Institute, Cary, NC, USA) was used for all statistical analyses.

## 3. Results

### 3.1. Patients’ Characteristics

**Figure 1:**
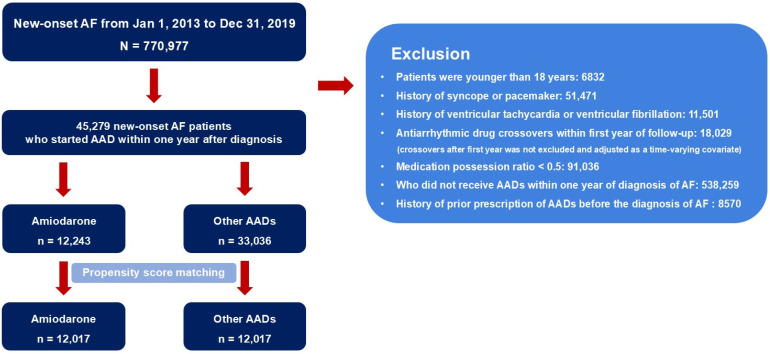
Flow of the study. AAD: antiarrhythmic drug; AF: atrial fibrillation.

During 1 January 2013, and 31 December 2019, we identified 770,977 new-onset AF patients. Exclusion criteria were as follows: (i) those who were prescribed with AADs before the diagnosis of new-onset AF (8570); (ii) those who did not receive AADs after diagnosis of new-onset AF (538,259); (iii) MPR < 0.5 (91,036); (iv) those who received multiple AADs or changed their type of AAD within one year after enrollment (18,029); (v) prior history of ventricular tachycardia or ventricular fibrillation (11,501); (vi) prior history of syncope or permanent pacemaker implantation (51,471); and (vii) those younger than 18 years (6823). Finally, a total of 45,279 people with new-onset AF were analyzed with 12,243 people in the amiodarone group and 33,036 people in the other-AAD group (Figure 1). Baseline characteristics are summarized in Table 1. People in the amiodarone group were older; had higher prevalence of diabetes mellitus, heart failure, myocardial infarction, chronic kidney disease, and stroke; and lower prevalence of dyslipidemia and thyroid disease (Table 1). We performed PSM to reduce bias due to various confounders, and matched variables were age, sex, alcohol consumption, regular exercise, income, diabetes, hypertension, dyslipidemia, heart failure, myocardial infarction, chronic kidney disease, hypo- or hyper-thyroidism, and stroke. After PSM, with 12,017 people for both groups, baseline characteristics did not show any clinically meaningful difference between amiodarone and other-AAD groups (Table 1). The MPR of first year after enrollment was 0.873 ± 0.160 and 0.911 ± 0.142 for amiodarone and other AAD groups, respectively.

### 3.2. Antiarrhythmic Drugs on All-Cause Death

**Figure 2:**
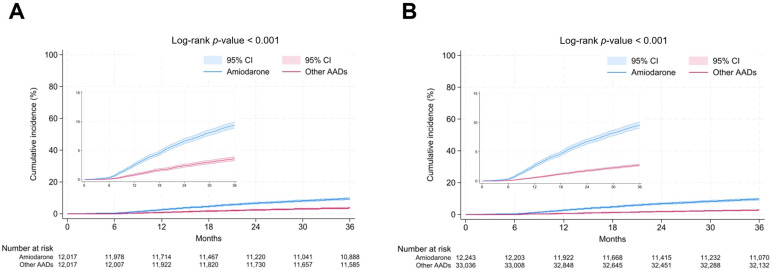
Cumulative incidence of all-cause death. Amiodarone was associated with significantly higher cumulative incidence of all-cause death compared with other AADs both in the whole cohort (**A**) and propensity-score-matched cohort (**B**). The occurrence of all-cause death was very low during first six months since medication possession ratio of equal or more than 0.5 (six months) was the inclusion criterion of this study. Shaded areas represent 95% confidence intervals. AAD: antiarrhythmic drug.

For 97,920 person × year follow-up, 904 death events occurred among 33,036 other-AAD users (incidence = 9.2). Among 12,243 amiodarone users, 1173 people died during 35,051 person × year follow-up (incidence = 33.5). Before multivariate adjustment, amiodarone users showed 3.6-fold increased risk of all-cause mortality (HR = 3.63; 95% CI = 3.33–3.95; *p* < 0.001; Table 2; Figure 2). After adjusting for age, sex, hypertension, diabetes mellitus, dyslipidemia, chronic kidney disease, heart failure, myocardial infarction, and thyroid disease, amiodarone use was associated with 2.9-fold increased risk of all-cause death (HR = 2.88; 95% CI = 2.64–3.15; *p* < 0.001; Table 2). When the type of AAD was considered as a time-varying covariate, amiodarone was associated with 3.2-fold increased risk of all-cause death (HR = 3.18; 95% CI = 2.42–4.18; *p* < 0.001; Table 2).

After propensity score matching, 432 and 1127 death events occurred in other AAD and amiodarone users, respectively (12,017 people for each group). Amiodarone was associated with 2.7-fold increased risk of all-cause death (HR = 2.68; 95% CI = 2.40–2.99; *p* < 0.001; Table 2; Figure 2) in non-adjusted analysis. In the multivariate model adjusting for age, sex, hypertension, diabetes mellitus, dyslipidemia, chronic kidney disease, heart failure, myocardial infarction, and thyroid disease, the risk of all-cause death was increased by 2.8-fold (HR = 2.75; 95% CI = 2.47–3.07; *p* < 0.001; Table 2). Hazard ratio for all-cause death of amiodarone was 3.56 (95% CI = 2.47–5.12; *p* < 0.001; Table 2) when the type of AAD was considered as a time-varying covariate. Further adjustment for the influence of catheter ablation of AF showed similar results (HR = 3.60; 95% CI = 3.00–4.32; *p* < 0.001; Table 2). The use of amiodarone in 14.6 patients (17.3 in PSM analysis) was associated with one excess mortality during a mean follow-up duration of 3 years (Table 3).

### 3.3. Subgroup Analysis

**Figure 3:**
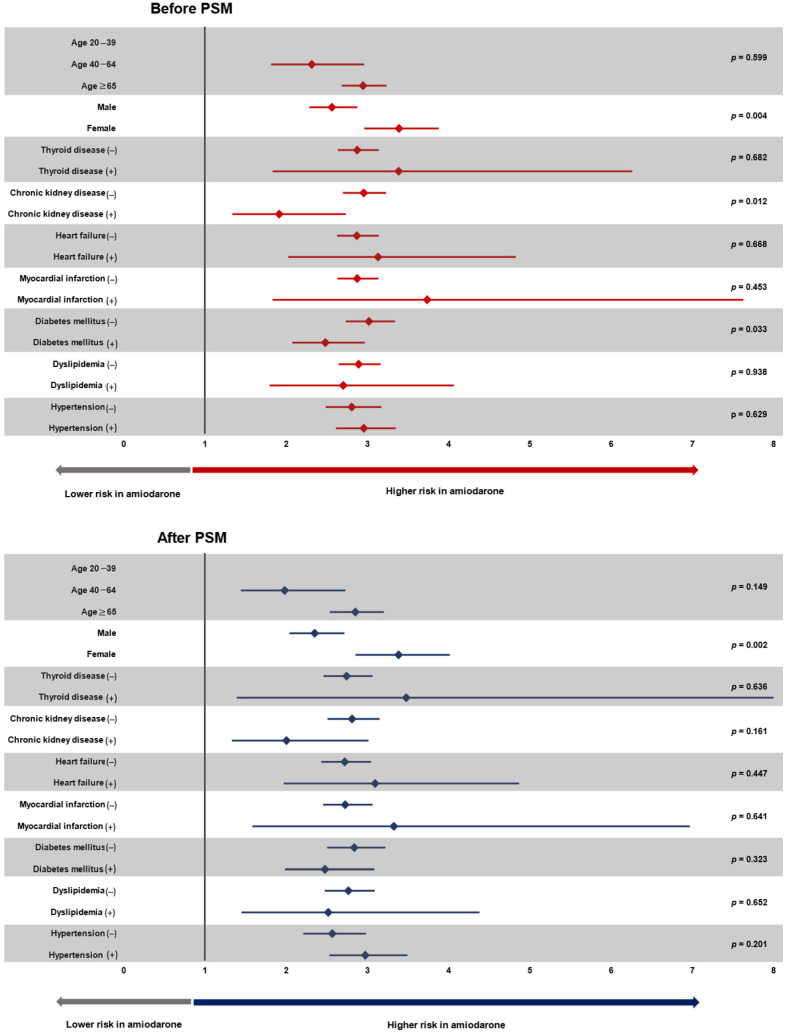
Subgroup analyses. Amiodarone was associated with significantly increased mortality across various subgroups in both before PSM and after PSM. Significant interaction was observed between the type of AAD and sex with women being more vulnerable to amiodarone. AAD: antiarrhythmic drug; HR: hazard ratio; PSM: propensity score matching. Hazard ratio was adjusted for age, sex, hypertension, diabetes mellitus, dyslipidemia, chronic kidney disease, heart failure, myocardial infarction, and thyroid disease.

The association between amiodarone use and increased risk of all-cause death was evaluated in various subgroups. The sex category showed a significant interaction, with women having more intense association between amiodarone use and increased risk of death (HR 3.38 in women and 2.56 in men; *p* value for interaction = 0.004; Figure 3 and Supplementary Table S4). This interaction was also shown in PSM analysis (Figure 3 and Supplementary Table S4). Significant interactions were also observed with chronic kidney disease and diabetes mellitus (Figure 3 and Supplementary Table S4). No interaction was observed with other covariates such as hypertension, dyslipidemia, myocardial infarction, heart failure, and thyroid disease (Figure 3 and Supplementary Table S4). In PSM analysis, only the sex category showed significant interaction with amiodarone use (HR was 3.38 and 2.35 for female and male participants, respectively; *p* value for interaction = 0.002; Figure 3 and Supplementary Table S4).

## 4. Discussion

The current study demonstrated the following: (i) amiodarone use as compared with other AADs was associated with a significant increase in overall mortality; (ii) women were more vulnerable to amiodarone use; and (iii) heart failure and myocardial infarction, traditional contraindications for most AADs such as class IC drugs or dronedarone, showed no significant interaction with amiodarone use. The strong points of the current study are the inclusion of new-onset AF rather than pre-existing AF patients and absence of follow-up losses except for emigrations. To ensure sufficient use of a certain AAD, MPR ≥ 0.5 within the first year after the initial prescription was used as an inclusion criteria, and mean MPR of analyzed patients during follow-up duration was greater than 0.9, suggesting that most patients analyzed in this study were sufficiently prescribed with AADs adequate for clinical comparison. In addition, due to exclusion of prevalent AF and prior use of AADs, included patients were not prescribed with AADs before enrollment excluding residual effect of prior prescription of AADs. Patients who changed their AAD during follow-up or had simultaneous use of multiple AADs were also excluded for the same purpose. We only included new-onset AF patients who started AAD within one year of diagnosis reflecting early rhythm control therapy, an emerging treatment strategy of AF.

### 4.1. Antiarrhythmic Drug in AF

AADs that act on ion channels are used for rhythm control of AF. The AFFIRM trial showed no mortality benefit of rhythm control strategy over rate control strategy [3]. Overall mortality was significantly increased in people equal or older than 65 years [3]. Furthermore, hospitalization events were significantly higher in the rhythm control group (80.1% vs. 73.0%; *p* < 0.001) [3]. Dronedarone, in the ATHENA trial, also showed no benefit in terms of all-cause death (5.0% in dronedarone vs. 6.0% in placebo; *p* = 0.18) [17]. However, it was associated with decreased risk of cardiovascular death (2.7% vs. 3.9%; *p* = 0.03) and first hospitalization due to cardiovascular events (32.4% vs. 42.6%; *p* < 0.001) [17]. The early rhythm control strategy in the EAST-AFNET 4 trial demonstrated a significant benefit in terms of cardiovascular mortality (HR = 0.72; 95% CI = 0.52–0.98) in addition to composite primary outcome (HR = 0.79; 95% CI = 0.66–0.94) [4]. The main antiarrhythmic drugs used to control AF were different among three studies: (i) amiodarone (37.5%) and sotalol (31.2%) in the AFFIRM trial [3]; (ii) dronedarone (100%) in the ATHENA trial [17]; and (iii) flecainide (35.9%) and amiodarone (19.6%) in the EAST-AFNET trial [4]. Whether these different clinical outcomes were due to the different type of AAD used in three trials is not fully known. Catheter ablation was also used as an initial treatment option in 8% of patients in the early rhythm control strategy group in the EAST-AFNET trial [4]. Early initiation of rhythm control treatment (enrolled 36 days after the first diagnosis of AF) is another different point of the trial [4].

The current study suggests that the use of amiodarone may increase the risk of all-cause mortality in new-onset AF patients compared with other AADs. Increased risk of all-cause death persisted after adjustment of various covariates such as age, sex, hypertension, diabetes mellitus, dyslipidemia, chronic kidney disease, heart failure, myocardial infarction, and thyroid disease. Propensity-score-matched analysis also demonstrated potential risk of amiodarone compared with other AADs. Other AADs such as propafenone, flecainide, or dronedarone are usually not recommended for people with heart failure or myocardial infarction, and amiodarone is spared for those patients. However, our study revealed no significant interactions between amiodarone use and heart failure/myocardial infarction in terms of overall death with amiodarone having a similar degree of increased mortality compared with other AADs in people with prior heart failure and myocardial infarction.

Prior studies revealed that clinical factors such as absence of heart failure, a small atrial size, recent-onset AF, and rapid heart rate were associated with a higher chance of spontaneous sinus conversion [18]. Considering possible association with increased mortality, amiodarone prescription can be limited to those who will likely achieve chemical cardioversion. Unnecessary prescription to AF patients who are unlikely to benefit from amiodarone should be avoided.

The sex category showed a significant interaction with amiodarone use, with women being more vulnerable to amiodarone than men. Clearance of amiodarone is low with an estimated elimination half-life of 30–180 days due to its lipophilic metabolite, desethylamiodarone, which causes the drug to accumulate in peripheral tissues with the highest concentrations observed in liver and lung, followed by pancreas and adipose tissue [19]. Since female patients have lower body weight than male patients, accumulation of amiodarone can be faster, and toxicities can be more severe than in male patients [20]. Amiodarone is lipophilic and can easily accumulate in fat tissues or tissues that have high fat composition, and higher fat composition rate in women can be another reason for the increased vulnerability in women to amiodarone [21,22]. Other variables such as hypertension, diabetes, chronic kidney disease, or heart failure did not show any interactions with amiodarone in PSM analysis. The reason for this disparity is unclear but clearance of amiodarone is minimally affected by renal function. Due to its high tissue-to-plasma-distribution ratio, differences in body tissue component and body weight in men and women might explain increased toxicities in women [23]. There is limited evidence that the presence of hypertension, diabetes, or heart failure can affect clearance of amiodarone.

### 4.2. Underlying Mechanism

A previous meta-analysis by Freemantle et al. reported that amiodarone is the most effective AAD to suppress AF [2,24]. However, clinical guidelines describe that non-amiodarone AADs should be considered first whenever possible due to extracardiac toxicity of amiodarone [2]. Our results are in line with the current guidelines by demonstrating significantly increased overall mortality in amiodarone users. The potential explanations for increased mortality associated with amiodarone use in our study include (i) systemic adverse effects, (ii) drug–drug interactions with non-vitamin K antagonist oral anticoagulant (NOAC), and (iii) selection bias. The original studies included in the prior meta-analysis were conducted in 1990s and 2000s when NOAC was not available in clinical practice, and amiodarone was not associated with increased mortality compared with other drugs in the study [20]. Amiodarone is known to increase plasma concentration of NOACs by 12% to 60% via moderate P-glycoprotein inhibition which can lead to increased risk of bleeding [25,26]. Our study enrolled patients when NOAC was available in clinical practice, and this difference might potentially explain increased mortality in amiodarone users. Another study by Qin et al. reported a 2.4-fold increased risk of all-cause death in amiodarone users which is a similar degree of increased mortality observed in our study [27]. The difference was mainly driven by non-cardiac mortality rather than cardiac mortality, suggesting extracardiac toxicities of amiodarone can actually lead to increased overall mortality [27]. Compared with a study by Qin et al. [27], our study had a significantly larger number of patients (2077 vs. 45,279), selectively included new-onset AF patients, and did not exclude patients with structural heart disease such as heart failure and myocardial infarction. Our study also has strong points in terms of absence of follow-up loss for all-cause death outcome due to the nature of K-NHIS, an exclusive single medical insurance system in South Korea.

### 4.3. Limitations

Several limitations exist in this study. First, our cohort is exclusively consisted of the East Asian population. Therefore, direct application of our data to other ethnic groups might not be feasible. Second, risk of coding errors can exist since our data are based on claim database despite validation of our coding strategies in various publications [15,16]. Third, selection biases can exist in our study. Non-amiodarone AADs are usually not prescribed in patients with heart failure or myocardial infarction. Therefore, amiodarone can be preferentially prescribed in such patients leading to a selection bias, although we adjusted such variables in our multivariate analysis. Unmeasured confounders can also exist, despite our efforts on vigorous multivariate adjustments and propensity score matching analysis. Fourth, we were not able to classify AF into paroxysmal and non-paroxysmal AF. Fifth, we were not able to obtain prescription history of oral anticoagulants, and their interaction with AADs could not be analyzed. Due to limitations of the K-NHIS database, we were not able to evaluate the cause of death. Sixth, we were not able to confirm the rhythm status of both groups during the follow-up period since this study was based on an insurance database. The burden of AF between the two groups might have been different during the follow-up which can influence the overall result of this study.

## 5. Conclusions

Amiodarone, compared with non-amiodarone AADs, was associated with increased risk of all-cause mortality in AAD-naive new-onset AF patients. Increased all-cause mortality associated with amiodarone was consistent throughout various subgroups including patients with prior heart failure and myocardial infarction. Significant interaction was observed with the sex category, with women being more vulnerable to amiodarone. A causal relationship cannot be confirmed since this was an observational study. Further randomized controlled trials are needed to evaluate the causal relationship between amiodarone and increased mortality in new-onset AF patients.

## Figures and Tables

**Table 1:** Baseline demographics.

	Before PSM				After PSM			
	AAD (+)	Amiodarone	Other AADs	*p* Value	Amiodarone	Other AADs	*p* Value	ASD
**n**	45,279	12,243	33,036		12,017	12,017		
**Age group**				<0.001			0.377	0.017
−39	1299	220 (1.8%)	1079 (3.3%)		220 (1.8%)	205 (1.7%)		
40–64	20,659	4555 (37.2%)	16,104 (48.7%)		4528 (37.7%)	4445 (37.0%)		
65–	23,321	7468 (61.0%)	15,853 (48.0%)		7269 (60.5%)	7367 (61.3%)		
**Sex**				<0.001			0.555	0.008
Male	27,606	7281 (59.5%)	20,325 (61.5%)		7158 (59.6%)	7113 (59.2%)		
Female	7673	4962 (40.5%)	12,711 (38.8%)		4859 (40.4%)	4904 (40.8%)		
Current smoker *				<0.001			<0.001	< 0.001
Non-smoker	15,564	3963 (32.4%)	11,601 (35.1%)		3931 (32.7%)	3936 (32.8%)		
Former-smoker	7395	1698 (13.9%)	5697 (17.2%)		1681 (14.0%)	1863 (15.5%)		
Current-smoker	4422	1216 (9.9%)	3206 (9.7%)		1208 (10.1%)	987 (8.2%)		
Missing value	17,898	5366 (43.8%)	12,532 (37.9%)		5197 (43.2%)	5231 (43.5%)		
Alcohol consumption *				<0.001			0.888	0.005
Non	15,778	4160 (34.0%)	11,618 (35.2%)		4115 (34.2%)	4087 (34.0%)		
Mild to moderate	9252	2105 (17.2%)	7147 (21.6%)		2096 (17.4%)	2112 (17.6%)		
Heavy	2319	603 (4.9%)	1716 (5.2%)		600 (5.0%)	579 (4.8%)		
Missing value	17,930	5375 (43.9%)	12,555 (38.0%)		5206 (43.3%)	5239 (43.6%)		
**Regular exercise**				<0.001			0.834	0.006
Yes	9884	2301 (18.8%)	7583 (23.0%)		4529 (37.7%)	4484 (37.3%)		
No	18,283	5366 (43.8%)	12,917 (39.1%)		2291 (19.1%)	2301 (19.1%)		
Missing value	17,112	4576 (37.4%)	12,536 (37.9%)		5197 (43.2%)	5232 (43.5%)		
**Income quartile**				<0.001			0.990	0.003
Q1 (lowest income)	9145	2800 (22.9%)	6345 (19.2%)		2734 (22.8%)	2741 (22.8%)		
Q2	6899	1904 (15.6%)	4995 (15.1%)		1869 (15.6%)	1872 (15.6%)		
Q3	9989	2707 (22.1%)	7282 (22.0%)		2648 (22.0%)	2626 (21.9%)		
Q4 (highest income)	18,541	4617 (37.7%)	13,924 (42.1%)		4551 (37.9%)	4571 (38.0%)		
Missing value	705	215 (1.8%)	490 (1.5%)		215 (1.8%)	207 (1.7%)		
**Diabetes mellitus**	7033	2234 (18.2%)	4799 (14.5%)	<0.001	2197 (18.3%)	2131 (17.7%)	0.268	0.015
**Hypertension**	19,479	5265 (43.0%)	14,214 (43.0%)	0.967	5182 (43.1%)	5194 (43.2%)	0.876	0.002
**Dyslipidemia**	3555	681 (5.6%)	2874 (8.7%)	<0.001	678 (5.6%)	674 (5.6%)	0.911	0.001
**Heart failure**	1037	603 (4.9%)	434 (1.3%)	<0.001	433 (3.6%)	406 (3.4%)	0.343	0.013
**Myocardial infarction**	547	316 (2.6%)	231 (0.7%)	<0.001	241 (2.0%)	205 (1.7%)	0.085	0.024
**Chronic kidney disease**	888	369 (3.0%)	519 (1.6%)	<0.001	305 (2.5%)	355 (3.0%)	0.848	0.003
**Hypo- or hyper-thyroidism**	1340	256 (2.1%)	1084 (3.3%)	<0.001	254 (2.1%)	244 (2.0%)	0.651	0.005
**Stroke**	2312	702 (5.7%)	1610 (4.9%)	<0.001	692 (5.8%)	680 (5.7%)	0.739	0.004
**Age**	64.5 ± 12.1	67.5 ± 12.1	63.4 ± 11.9	<0.001	67.3 ± 12.2	66.2 ± 11.5	<0.001	0.096
Fasting glucose (mg/dL) *	105 ± 26	107.7 ± 30.3	104.1 ± 24.3	<0.001	107.7 ± 30.2	105.4 ± 25.8	<0.001	
Body mass index (kg/m^2^) *	24.9 ± 3.3	25.1 ± 3.5	24.9 ± 3.3	<0.001	25.1 ± 3.6	24.8 ± 3.3	<0.001	
Waist circumference (cm) *	85.4 ± 9	86.2 ± 9.3	85.1 ± 8.8	<0.001	86.2 ± 9.3	85.2 ± 8.9	<0.001	
Systolic blood pressure (mmHg) *	127.4 ± 15.3	128.4 ± 16	127 ± 15.1	<0.001	128.4 ± 16	128.1 ± 15.3	0.346	
Diastolic blood pressure (mmHg) *	77.8 ± 10.2	78 ± 10.6	77.7 ± 10.1	0.014	78 ± 10.6	77.7 ± 10.1	0.029	
eGFR *	83 ± 26	80.1 ± 25.8	84 ± 26	<0.001	80.2 ± 25.8	81.9 ± 23.8	<0.001	
Total cholesterol (mg/dL) *	188.3 ± 43.5	185.6 ± 46.9	189.2 ± 42.3	<0.001	185.7 ± 46.9	187.1 ± 49.5	0.090	
**Catheter ablation**	5053 (11.2%)	885 (7.2%)	4168 (12.6%)	<0.001	879 (7.3%)	1333 (11.1%)	<0.001	
**MPR (mean)**	0.90 ± 0.15	0.87 ± 0.16	0.91 ± 0.14	<0.001	0.87 ± 0.16	0.91 ± 0.14	<0.001	

* Self questionnaire and laboratory data were available for people who underwent nationwide health screening. Propensity score matching was performed with covariates including age, sex, alcohol consumption, regular exercise, income, diabetes, hypertension, dyslipidemia, heart failure, myocardial infarction, chronic kidney disease, hypo- or hyper-thyroidism, and stroke. AAD: antiarrhythmic drug; ASD: absolute standardized difference; eGFR: estimated glomerular filtration rate; PSM: propensity score matching.

**Table 2:** Amiodarone vs. other AADs for all-cause death.

	n	Event Number (All-Cause Death)	Duration (Person × Year)	Incidence	Non-Adjusted	Model 1	Model 2	Model 3	Model 4	Model 5
**Before PSM**										
other AADs	33,036	904	97,920	9.2	reference	reference	reference	reference	reference	reference
Amiodarone	12,243	1173	35,051	33.5	3.63 (3.33–3.95)	3.09 (2.84–3.37)	2.88 (2.64–3.15)	2.53 (2.23–2.87)	3.18 (2.42–4.18)	3.81 (3.30–4.40)
**After PSM**										
other AADs	12,017	432	35,473	12.2	reference	reference	reference	reference	reference	reference
Amiodarone	12,017	1127	34,442	32.7	2.68 (2.40–2.99)	2.74 (2.46–3.06)	2.75 (2.47–3.07)	2.38 (2.03–2.79)	3.56 (2.47–5.12)	3.60 (3.00–4.32)

Incidence is per 1000 person × year follow-up. Model 1: age and sex. Model 2: age, sex, hypertension, diabetes mellitus, dyslipidemia, chronic kidney disease, heart failure, myocardial infarction, and thyroid disease. Model 3: model 2 + alcohol, smoking, body mass index, regular exercise, estimated glomerular filtration rate, and total cholesterol. Model 4: model 2 + AAD as a time-varying covariate Model 5: model 4 + catheter ablation for atrial fibrillation during the screening and follow-up period. AAD: antiarrhythmic drug; PSM: propensity score matching.

**Table 3:** Benefits of non-amiodarone AADs.

	n	Event Number	Event Rate	Absolute Risk Reduction	Number Needed to Treat
**Before PSM**					
other AADs	33,036	904	0.027	0.069	14.61
Amiodarone	12,243	1173	0.096		
	
**After PSM**					
other AADs	12,017	432	0.036	0.058	17.29
Amiodarone	12,017	1127	0.094		
	

AADs: antiarrhythmic drugs.

## Data Availability

The data underlying this article are available in the article and in its online Supplementary Material.

## Supplementary Materials

The following supporting information can be downloaded at: https://www.mdpi.com/article/10.3390/jcm14041168/s1, Table S1: ICD-10 codes for atrial fibrillation; Table S2: Drugs analyzed in this study; Table S3: ICD-10 codes for various medical conditions; Table S4: Subgroup analysis.

## Abbreviations

AAD: antiarrhythmic drug; AF: atrial fibrillation; CI: confidence interval; HR: hazard ratio; ICD-10: International Classification of Disease, 10th edition; K-NHIS: Korean National Health Insurance Service; MPR: medication possession ratio; PSM: propensity score matching.

## References

[1] Kim Y.G., Choi J.-I., Kim H.-J., Min K., Choi Y.Y., Shim J., Son H.S., Kim Y.-H. (2022). A Watch-Type Electrocardiography Is a Reliable Tool for Detecting Paroxysmal Cardiac Arrhythmias. J. Clin. Med..

[2] Hindricks G., Potpara T., Dagres N., Arbelo E., Bax J.J., Blomström-Lundqvist C., Boriani G., Castella M., Dan G.-A., Dilaveris P.E. (2021). 2020 ESC Guidelines for the diagnosis and management of atrial fibrillation developed in collaboration with the European Association for Cardio-Thoracic Surgery (EACTS) The Task Force for the diagnosis and management of atrial fibrillation of the European Society of Cardiology (ESC) Developed with the special contribution of the European Heart Rhythm Association (EHRA) of the ESC. Eur. Heart J..

[3] Van Gelder I.C., Hagens V.E., Bosker H.A., Kingma J.H., Kamp O., Kingma T., Said S.A., Darmanata J.I., Timmermans A.J., Tijssen J.G. (2002). A comparison of rate control and rhythm control in patients with recurrent persistent atrial fibrillation. N. Engl. J. Med..

[4] Kirchhof P., Camm A.J., Goette A., Brandes A., Eckardt L., Elvan A., Fetsch T., van Gelder I.C., Haase D., Haegeli L.M. (2020). Early rhythm-control therapy in patients with atrial fibrillation. N. Engl. J. Med..

[5] Roy D., Talajic M., Dorian P., Connolly S., Eisenberg M.J., Green M., Kus T., Lambert J., Dubuc M., Gagné P. (2000). Amiodarone to prevent recurrence of atrial fibrillation. N. Engl. J. Med..

[6] Singh B.N., Singh S.N., Reda D.J., Tang X.C., Lopez B., Harris C.L., Fletcher R.D., Sharma S.C., Atwood J.E., Jacobson A.K. (2005). Amiodarone versus sotalol for atrial fibrillation. N. Engl. J. Med..

[7] e1 AFADSI (2003). Maintenance of sinus rhythm in patients with atrial fibrillation: An AFFIRM substudy of the first antiarrhythmic drug. J. Am. Coll. Cardiol..

[8] Pearce E.N., Farwell A.P., Braverman L.E. (2003). Thyroiditis. New Engl. J. Med..

[9] Goldschlager N., Epstein A.E., Naccarelli G.V., Olshansky B., Singh B., Collard H.R., Murphy E. (2007). A practical guide for clinicians who treat patients with amiodarone: 2007. Heart Rhythm.

[10] Olshansky B., Sami M., Rubin A., Kostis J., Shorofsky S., Slee A., Greene H.L., NHLBI AFFIRM Investigators (2005). Use of amiodarone for atrial fibrillation in patients with preexisting pulmonary disease in the AFFIRM study. Am. J. Cardiol..

[11] Sanyal A.J. (2002). AGA technical review on nonalcoholic fatty liver disease. Gastroenterology.

[12] Zimetbaum P. (2007). Amiodarone for Atrial Fibrillation. N. Engl. J. Med..

[13] Køber L., Torp-Pedersen C., McMurray J.J., Gøtzsche O., Lévy S., Crijns H., Amlie J., Carlsen J. (2008). Increased mortality after dronedarone therapy for severe heart failure. N. Engl. J. Med..

[14] Echt D.S., Liebson P.R., Mitchell L.B., Peters R.W., Obias-Manno D., Barker A.H., Arensberg D., Baker A., Friedman L., Greene H.L. (1991). Mortality and morbidity in patients receiving encainide, flecainide, or placebo: The Cardiac Arrhythmia Suppression Trial. N. Engl. J. Med..

[15] Kim Y.G., Han K.-D., Kim D.Y., Choi Y.Y., Choi H.Y., Roh S.-Y., Shim J., Kim J.S., Choi J.-I., Kim Y.-H. (2021). Different Influence of Blood Pressure on New-Onset Atrial Fibrillation in Pre-and Postmenopausal Women: A Nationwide Population-Based Study. Hypertension.

[16] Kim Y.G., Han K.-D., Choi J.-I., Choi Y.Y., Choi H.Y., Boo K.Y., Kim D.Y., Lee K.-N., Shim J., Kim J.-S. (2021). Non-genetic risk factors for atrial fibrillation are equally important in both young and old age: A nationwide population-based study. Eur. J. Prev. Cardiol..

[17] Hohnloser S.H., Crijns H.J., Van Eickels M., Gaudin C., Page R.L., Torp-Pedersen C., Connolly S.J. (2009). Effect of dronedarone on cardiovascular events in atrial fibrillation. N. Engl. J. Med..

[18] Mariani M.V., Pierucci N., Piro A., Trivigno S., Chimenti C., Galardo G., Miraldi F., Vizza C.D. (2022). Incidence and determinants of spontaneous cardioversion of early onset symptomatic atrial fibrillation. Medicina.

[19] Brien J.F., Jimmo S., Brennan F.J., Ford S.E., Armstrong P.W. (1987). Distribution of amiodarone and its metabolite, desethylamiodarone, in human tissues. Can. J. Physiol. Pharmacol..

[20] Kim H.-L., Seo J.-B., Chung W.-Y., Kim S.-H., Kim M.-A., Zo J.-H. (2014). The incidence and predictors of overall adverse effects caused by low dose amiodarone in real-world clinical practice. Korean J. Intern. Med..

[21] Adams P., Holt D., Storey G., Morley A., Callaghan J., Campbell R. (1985). Amiodarone and its desethyl metabolite: Tissue distribution and morphologic changes during long-term therapy. Circulation.

[22] Lafuente-Lafuente C., Alvarez J.C., Leenhardt A., Mouly S., Extramiana F., Caulin C., Funck-Brentano C., Bergmann J.F. (2009). Amiodarone concentrations in plasma and fat tissue during chronic treatment and related toxicity. Br. J. Clin. Pharmacol..

[23] Freedman M.D., Somberg J.C. (1991). Pharmacology and pharmacokinetics of amiodarone. J. Clin. Pharmacol..

[24] Freemantle N., Lafuente-Lafuente C., Mitchell S., Eckert L., Reynolds M. (2011). Mixed treatment comparison of dronedarone, amiodarone, sotalol, flecainide, and propafenone, for the management of atrial fibrillation. Europace.

[25] Gnoth M.J., Buetehorn U., Muenster U., Schwarz T., Sandmann S. (2011). In vitro and in vivo P-glycoprotein transport characteristics of rivaroxaban. J. Pharmacol. Exp. Ther..

[26] Steffel J., Collins R., Antz M., Cornu P., Desteghe L., Haeusler K.G., Oldgren J., Reinecke H., Roldan-Schilling V., Rowell N. (2021). 2021 European Heart Rhythm Association practical guide on the use of non-vitamin K antagonist oral anticoagulants in patients with atrial fibrillation. Europace.

[27] Qin D., Leef G., Alam M.B., Rattan R., Munir M.B., Patel D., Khattak F., Adelstein E., Jain S.K., Saba S. (2015). Mortality risk of long-term amiodarone therapy for atrial fibrillation patients without structural heart disease. Cardiol. J..

