# Supplementary material for: Increased Mortality Associated with Amiodarone Compared to Other Antiarrhythmic Drugs in New-Onset Atrial Fibrillation
Source: J Clin Med. 2025 Feb 11;14(4):1168. doi: 10.3390/jcm14041168 (PMC11856129; doi:10.3390/jcm14041168)
Supplement: Supplementary file 1 [file jcm-14-01168-s001.zip › jcm-3413588-supplementary.pdf]

Increased Mortality Associated with Amiodarone Compared to Other Antiarrhythmic Drugs in New-onset Atrial Fibrillation.

Yun Gi Kim, MD, PhD[1], Hyoung Seok Lee, MD[1], Hoseob Kim, MPH[2], Mina Kim, MS[2], Joo Hee Jeong, MD[1], Yun Young Choi, MD, PhD[1], Jaemin Shim, MD, PhD[1], Jong-Il Choi, MD, PhD, MHS, MSc[1]* and Young-Hoon Kim, MD, PhD[1]

[1]Division of Cardiology, Department of Internal Medicine, Korea University College of Medicine and Korea University Anam Hospital, Seoul, Republic of Korea

[2]Department of Data Science, Hanmi Pharm. Co., Ltd

*Address for correspondence: Jong-Il Choi, MD, PhD, MHS, MSc, FESC

[1]Division of Cardiology, Department of Internal Medicine, Korea University College of Medicine and Korea University Anam Hospital, Seoul, Republic of Korea

73 Goryeodae-ro, Seongbuk-gu, Seoul 02841, Republic of Korea

Tel: 82-2-920-5445 / Fax: 82-2-927-1478 / E-mail: jongilchoi@korea.ac.kr

Running title: Amiodarone and increased mortality

Disclosure: The authors have nothing to disclose.

Total word count: 767

Yun Gi Kim and Hyoung Seok Lee contributed equally to this work.

Supplementary Table S1. ICD-10 codes for atrial fibrillation.

Code	Name
I48.0	Paroxysmal atrial fibrillation
I48.1	Persistent atrial fibrillation
I48.2	Chronic atrial fibrillation
I48.9	Atrial fibrillation and atrial flutter, unspecified

ICD-10: international classification of diseases, 10th edition.

Supplementary Table S2. Drugs analyzed in this study.

Type	Code	Name
Flecainide	501902BIJ	Flecainide 150mg
	501901BIJ	Flecainide 50mg
	159301ATB	Flecainide acetate 100mg
	159331BIJ	Flecainide acetate150mg (10mg/mL)
	159302ATB	Flecainide acetate 50mg
	159330BIJ	Flecainide acetate 50mg (10mg/mL)
Amiodarone	107402BIJ	Amiodarone HCl 150mg
	107430BIJ	Amiodarone hydrochloride 0.15g (50mg/mL)
	107401ATB	Amiodarone hydrochloride 0.2g
Pilsicainide	502101ACH	Pilsicainide HCl 25mg
	502102ACH	Pilsicainide HCl 50mg
	502101ACH	Pilsicainide hydrochloride 25mg
	502102ACH	Pilsicainide hydrochloride 50mg
Propafenone	219501ATB	Propafenone HCl 150mg
	219503ACR	Propafenone HCl 225mg
	219502ATB	Propafenone HCl 300mg
	219504ACR	Propafenone HCl 325mg
	219505ACR	Propafenone HCl 425mg
	219501ATB	Propafenone hydrochloride 0.15g
	219503ACR	Propafenone hydrochloride 0.225g
	219504ACR	Propafenone hydrochloride 0.325g
	219502ATB	Propafenone hydrochloride 0.3g
	219505ACR	Propafenone hydrochloride 0.425g
Sotalol	230401ATB	Sotalol HCl 160mg
	230402ATB	Sotalol HCl 40mg
	230402BIJ	Sotalol HCl 40mg
	230403ATB	Sotalol HCl 80mg
Dronedarone	597401ATB	Dronedarone 0.4g

Supplementary Table S3. ICD-10 codes for various medical conditions.

Covariates	Code	Name
Myocardial infarction	I21	Acute myocardial infarction
	I22	Subsequent myocardial infarction
	I23	Certain current complications following acute myocardial infarction
Hypertension	I10	Essential (primary) hypertension
	I11	Hypertensive heart disease
	I12	Hypertensive renal disease
	I13	Hypertensive heart and renal disease
	I15	Secondary hypertension
Diabetes mellitus	E10	Type 1 diabetes mellitus
	E11	Type 2 diabetes mellitus
	E12	Malnutrition-related diabetes mellitus
	E13	Other specified diabetes mellitus
	E14	Unspecified diabetes mellitus
Dyslipidemia	E78	Disorders of lipoprotein metabolism and other lipidemias
Hypo- or hyper-thyroidism	E03	Other hypothyroidism
	E05	Thyrotoxicosis [hyperthyroidism]
Stroke	I60	Subarachnoid hemorrhage
	I61	Intracerebral hemorrhage
	I62	Other nontraumatic intracranial hemorrhage
	I63	Cerebral infarction
	I64	Stroke, not specified as hemorrhage or infarction
Heart failure	I50	Heart failure
Chronic kidney disease	N18	Chronic kidney disease
	N19	Unspecified kidney failure
	Z49	Persons encountering health services for care involving dialysis
	Z99.2	Dependence on renal dialysis

ICD-10: international classification of diseases, 10th edition.

Supplementary Table S4. Subgroup analysis.

	**Before PSM**		**After PSM**	
	**Hazard ration (95% confidence interval)**	**p value for interaction**	**Hazard ration (95% confidence interval)**	**p value for interaction**
**Age group**		0.599		0.149
20 – 39	NA	NA	NA	NA
40 – 64	2.317 (1.815 – 2.958)	< 0.001	1.982 (1.439 – 2.729)	< 0.001
65 –	2.947 (2.683 – 3.236)	< 0.001	2.850 (2.536 – 3.203)	< 0.001
**Sex**		0.004		0.002
Male	2.561 (2.283 – 2.872)	< 0.001	2.353 (2.039 – 2.716)	< 0.001
Female	3.388 (2.959 – 3.878)	< 0.001	3.383 (2.852 – 4.013)	< 0.001
**Thyroid disease**		0.682		0.636
No	2.875 (2.633 – 3.140)	< 0.001	2.743 (2.457 – 3.063)	< 0.001
Yes	3.385 (1.831 – 6.260)	< 0.001	3.478 (1.391 – 8.697)	0.0077
**Chronic kidney disease**		0.012		0.161
No	2.953 (2.700 – 3.230)	< 0.001	2.811 (2.508 – 3.149)	< 0.001
Yes	1.912 (1.337 – 2.733)	< 0.001	2.002 (1.330 – 3.015)	< 0.001
**Heart failure**		0.668		0.447
No	2.871 (2.626 – 3.138)	< 0.001	2.722 (2.431 – 3.047)	< 0.001

Yes	3.129 (2.028 – 4.828)	< 0.001	3.094 (1.967 – 4.866)	< 0.001
**Myocardial infarction**		0.453		0.641
No	2.872 (2.630 – 3.136)	< 0.001	2.742 (2.455 – 3.063)	< 0.001
Yes	3.736 (1.831 – 7.626)	< 0.001	3.323 (1.584 – 6.969)	0.002
**Diabetes mellitus**		0.033		0.323
No	3.020 (2.734 – 3.337)	< 0.001	2.839 (2.502 – 3.221)	< 0.001
Yes	2.482 (2.076 – 2.967)	< 0.001	2.475 (1.986 – 3.084)	< 0.001
**Dyslipidemia**		0.938		0.652
No	2.893 (2.646 – 3.163)	< 0.001	2.765 (2.473 – 3.091)	< 0.001
Yes	2.701 (1.795 – 4.065)	< 0.001	2.518 (1.447 – 4.381)	0.001
**Hypertension**		0.629		0.201
No	2.806 (2.484 – 3.171)	< 0.001	2.569 (2.212 – 2.983)	< 0.001
Yes	2.956 (2.611 – 3.348)	< 0.001	2.974 (2.532 – 3.493)	< 0.001

NA: not applicable; PSM: propensity-score matching. Hazard ratios were adjusted for age, sex, hypertension, diabetes mellitus, dyslipidemia, chronic kidney disease, heart failure, myocardial infarction, and thyroid disease.
